# Temperature and Magnetic Field Driven Modifications in the I-V Features of Gold-DNA-Gold Structure

**DOI:** 10.3390/s141019229

**Published:** 2014-10-15

**Authors:** Nadia Mahmoudi Khatir, Zulkurnain Abdul-Malek, Seyedeh Maryam Banihashemian

**Affiliations:** 1 Institute of High Voltage and High Current, Faculty of Electrical Engineering, Universiti Teknologi Malaysia, 81310 Johor Bahru, Malaysia; 2 Low Dimensional Materials Research Centre, Department of Physics, University of Malaya, Kuala Lumpur 50603, Malaysia; E-Mail: smb137@yahoo.com

**Keywords:** sensor, GDG structure, DNA, I-V curve, temperature, magnetic field

## Abstract

The fabrication of Metal-DNA-Metal (MDM) structure-based high sensitivity sensors from DNA micro-and nanoarray strands is a key issue in their development. The tunable semiconducting response of DNA in the presence of external electromagnetic and thermal fields is a gift for molecular electronics. The impact of temperatures (25–55 °C) and magnetic fields (0–1200 mT) on the current-voltage (I-V) features of Au-DNA-Au (GDG) structures with an optimum gap of 10 μm is reported. The I-V characteristics acquired in the presence and absence of magnetic fields demonstrated the semiconducting diode nature of DNA in GDG structures with high temperature sensitivity. The saturation current in the absence of magnetic field was found to increase sharply with the increase of temperature up to 45 °C and decrease rapidly thereafter. This increase was attributed to the temperature-assisted conversion of double bonds into single bond in DNA structures. Furthermore, the potential barrier height and Richardson constant for all the structures increased steadily with the increase of external magnetic field irrespective of temperature variations. Our observation on magnetic field and temperature sensitivity of I-V response in GDG sandwiches may contribute towards the development of DNA-based magnetic sensors.

## 1. Introduction

Recently, the tunable electrical and magnetic properties of biomaterials have become attractive due to their usefulness in the development of efficient miniaturized electronic devices [1,2]. The advent of DNA material-based nanodevices created via bottom-ups [3] approaches has imparted further impetus in materials research. Meanwhile, the semiconducting properties of DNA-based devices in the presence of external electric and magnetic fields has received special attention for their prospective use as magnetic sensors [4–8]. The path-breaking discovery of the DNA structure by Watson and Crick stimulated rapid developments in the fields of biology, genetics, medicine [9] and nanoelectronics [10,11]. Previously, DNA-based biosensors were employed for gene analysis, detection of genetic disorders and tissue matching [12–14] using magnetic particle [15] and temperature sensing [16]. These are complementary with our observed results, which report on the controlled behaviour of the magnetic field and temperature dependent electronic properties of DNA. DNA molecules, which contain sugar-phosphate groups and four nucleotides-adenine (A), guanine (G), thymine (T) and cytosine (C)-are found to be responsible for multi-faceted properties [17,18]. Furthermore, these nucleotides fit closely to form very effective hydrogen bonds with each other, where A is always paired with T and G is bonded with C [19]. It is the high flexibility of the DNA structures that ensures the possibility of tuning electronic properties through the application of external fields. Despite some dedicated efforts, issues related to the impact of temperature on the current transport in DNA structures in the presence of magnetic fields remain debatable.

Lately, DNA structure-based nanomachines, nanotemplates and nanoelectronics (using one-dimensional molecular wires) have materialized [20–23]. The research interest in DNA is enhanced due to its effectiveness in nanoelectronic devices, either as a template for assembling nanocircuits or as a piece of such circuits. Definitely, the occurrence of a practical conducting variant of DNA has influenced advancements in nanotechnology immensely [24–29]. Over the years, numerous issues related to the rectifier characteristics have improved considerably in molecular electronics. Much of these shortcomings are related to the work function incongruity between two different metals or the metal-molecule interfaces, the placement of the chromophore between the two metal electrodes and the molecular orbitals acting as different sources that create asymmetric I-V features [30,31]. The founding work of Aviram and Ratner in 1974 demonstrated the electronic rectification of DNA. They fabricated a simplistic electronic device based on a single organic molecule consisting of a donor-sigma bond-acceptor [32]. Research on magnetic biosensors and magnetoresistive biochips is continually progressing due to their potential as specific bio-systems in achieving great sensitivity, low delay time and high throughput [33–37].

In this work the temperature and magnetic field assisted I-V characteristics of GDG structures are examined. The feasibility of using these structures for magnetic and thermal field detection or sensing is demonstrated. The temperature-driven enhancement in the potential barrier and saturation current of GDG sandwiches indeed displayed efficient sensing prospects. The results are analyzed, compared and the mechanism of sensitivity enhancement is understood.

## 2. Experimental Section

The *Boesenbergia rotunda* plant was chosen to partially extract DNA strands of which the sequence and relative percent of T, A, C and G are summarized in Table 1. Analytical grade chemicals of high purity (Sigma Aldrich) such as HCl, NH_3_, H_2_O_2_, HF and acetone were used. Cr (99.999% purity) and Au (99.999% purity) are used for evaporation and magnetron sputtering (Kurt Company, Hudson Valley, PA, USA). The AZ 1500 series photoresist and developer from Sigma Aldrich (Selangor, Malaysia) were employed.

A p-type Si(100) wafer of dimension 1 cm × 1 cm having resistivity of 1 to 10–20 Ω-cm (MEMC Electronic Materials, Selangor, Malaysia) together with a 1000 nm thick SiO_2_ layer was used as the substrate. They were thoroughly cleaned using the reaction chemical agents (RCA) method. The substrate is boiled for 10 min in a solution made of NH_3_, H_2_O_2_ and H_2_O (ratio 1:1:6) followed by another 10 min in a solution composed of HCl, H_2_O_2_ and H_2_O (ratio 1:1:6). The native oxide appears on the substrate front surface is removed using HF and H_2_O solution (ratio 1:10) before being rinsed in deionized water for 30 s. Subsequently, UV-lithography with a designed mask is used to deposit the AZl512 photoresist. Finally, DC magnetron sputtering (NTI Nanofilm, Singapore) and thermal evaporation techniques (Edward Auto 306, West Sussex, UK) are used to deposit Cr and Au layers of thicknesses of 90 and 150 nm, respectively. A DNA solution of suitable concentration (0.01 mg/mL) is used to dilute it. The solution is allowed to flow along the Au surface using a microsyringe. The I-V characteristics of the fabricated GDG structure in the absence and presence of magnetic fields at different temperatures are measured by a semiconductor analyzer (SA) (SMU-236, Keithley, OH, USA).

**Figure 1.**
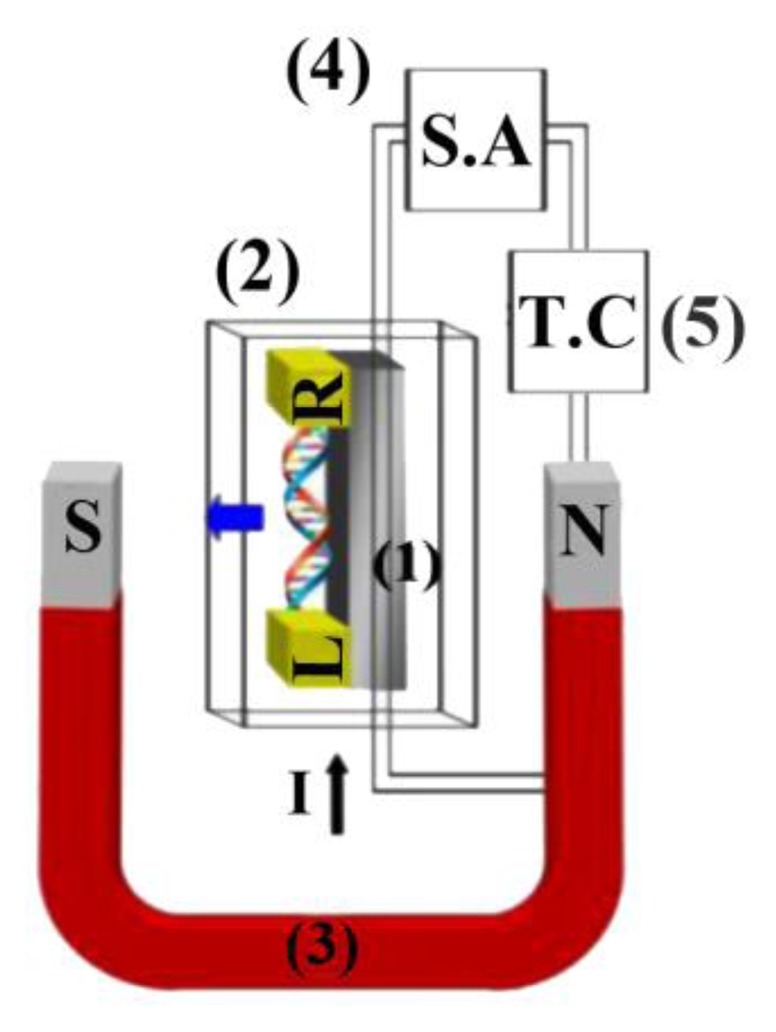
Experimental design with labels: (1) chip holder and connection in a Dewar, (2) cryostat system, (3) magnetic field generator and detector, (4) semiconductor analyzer for I-V measurement and (5) thermal controller.

Figure 1 illustrates the experimental set-up, consisting of a chip holder, cryostat system with a large stainless steel container surrounding the vacuum holder and superconducting magnets furnishing a super-cool and vacuum environment (VPF-100, Janis, MA, USA), a 1000 turn coil magnetic field generator, SA and thermal controller (TC).

## 3. Results and Discussion

**Figure 2.**
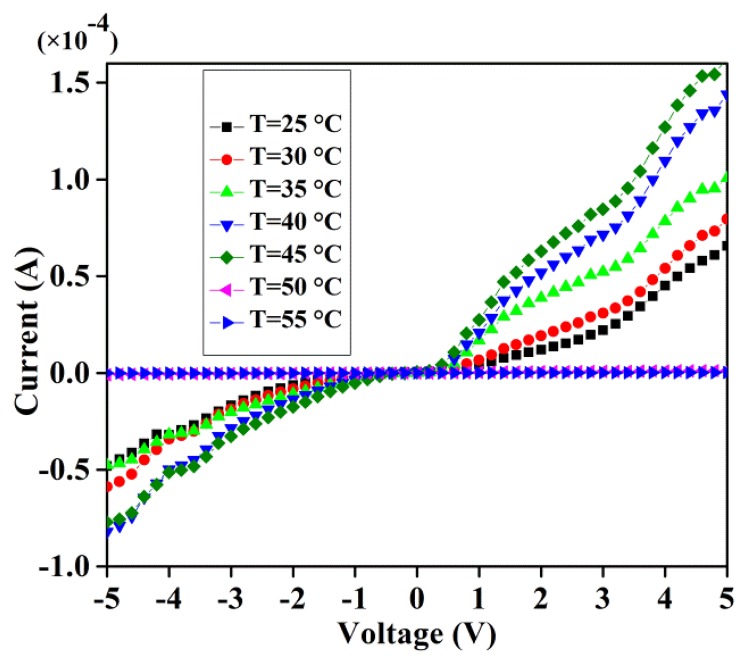
I-V curves of GDG structure at various temperatures in the absence of magnetic field.

The sample is placed in a cryostat under the influence of an external magnetic field generated by an electromagnet and connected to the SA via TC. The recorded I-V curves for the GDG structure in the presence of various magnetic fields and temperatures reveal rectifying behavior under forward bias. As illustrated in Figure 2, in the absence of magnetic field the current is increased exponentially with low threshold voltage as the temperature is raised up to 45 °C and decreases thereafter.

Thus, DNA strands in the GDG structures act as a semiconductor equivalent back-to-back diode. For a given specified voltage and with increasing temperature the reverse bias current is reduced compared to the forward bias region showing a changing diode threshold voltage.

**Figure 3.**
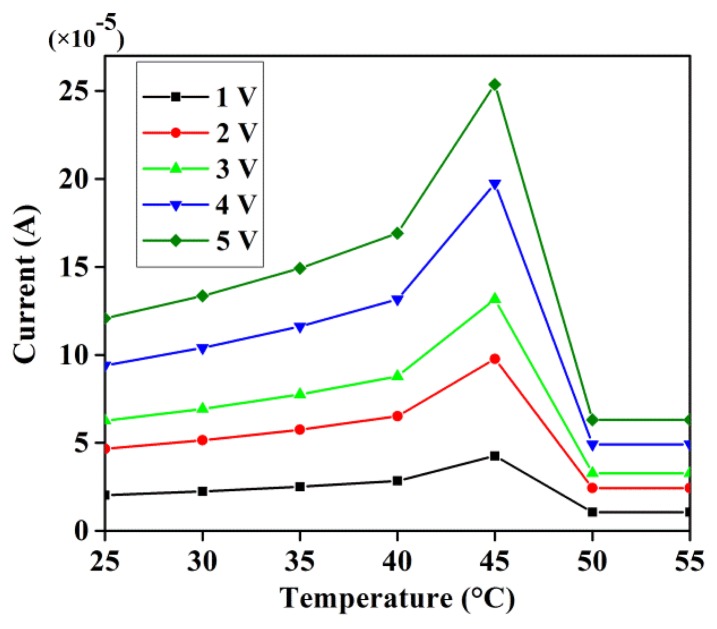
Temperature dependent current of GDG structure at different voltages in the absence of magnetic field.

Figure 3 displays the temperature-dependent current response at varying bias voltages. The current is found to increase steadily with the increase of temperature up to 45 °C and suddenly decrease beyond that point, irrespective of voltages. This behaviour is attributed to the structural modifications in the material from its original state, where the hydrogen bonds between the base pairs of the DNA macromolecular structure break with the increase of temperature [38]. This temperature-driven conversion of double strands into single strands via bond ruptures results in an increase in the junction resistance and thereby a decrease in the current. This breakage appears prominent at higher temperatures and the current is reduced significantly. Following the metal-semiconductor contact equation of current-voltage-temperature, the melting temperature of the DNA used is calculated via the BioMath computer program. The melting point of DNA molecules is the temperature at which half the DNA molecules are denatured [39]. Table 1 lists the melting point, length and percentage of G and C in the DNA. The melting point of DNA of length 567 nm with 58% G-C is estimated to be 87.5 °C. Our experiment revealed that the DNA sample began to denature at 45 °C, which is responsible for the decrease in current. Conversely, the constant current at 50 °C indicated that the DNA was denatured enough to make the conduction impossible. Accordingly, conduction was stopped before the estimated melting point was reached due to the initiation of the denaturation process. It can be concluded that double stranded DNA has good conductivity below 55 °C and appeared effectively nonconductive beyond this temperature. The melting point for shorter DNA strands is lower than that of normal sized DNA strands. The presence of higher percentage of guanine and cytosine is found to increase the melting point [40].

**Figure 4.**
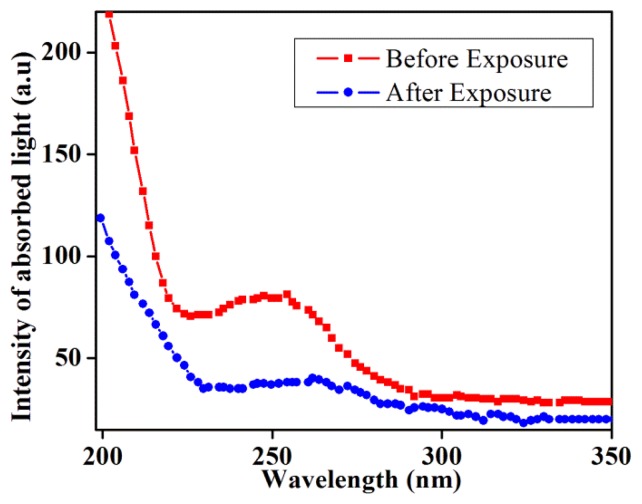
The UV-VIS spectrum of DNA before and after exposure to electromagnetic fields.

The concentration of DNA after the field exposure calculated from the UV-Vis spectra as displayed in Figure 4 showed a considerable decline. Concentrations before and after exposure were found to be 0.3987 and 0.1947 (ng/nL), respectively. The intensity of absorbed light after exposure with increasing temperature also revealed a significant reduction. Generally, the absorption of light increased with the increase of DNA temperature. However, the evidence seems to indicate decreasing light absorption. DNA absorbs UV-light due to its aromatic rings and the decrease of these rings due to the change in structure caused a decrease in the absorbed light intensity.

**Figure 5.**
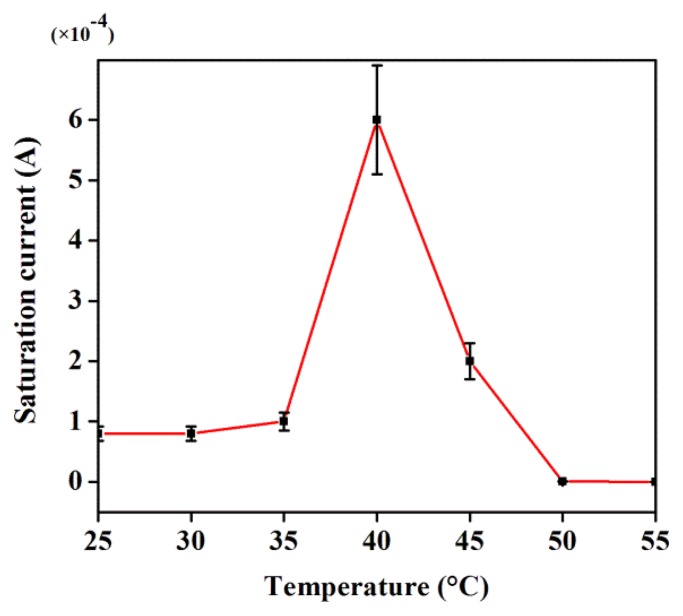
Temperature dependent saturation current in the absence of external magnetic field.

Figure 5 shows the temperature dependence of the saturation current without applied magnetic field. The saturation current exhibits a steady increase with the increase of temperature up to 45 °C and a sudden drop thereafter. This observation is interpreted in terms of the structural alterations in DNA where double bonds get converted into single bonds and these results in an augmentation in the junction resistance.

**Figure 6.**
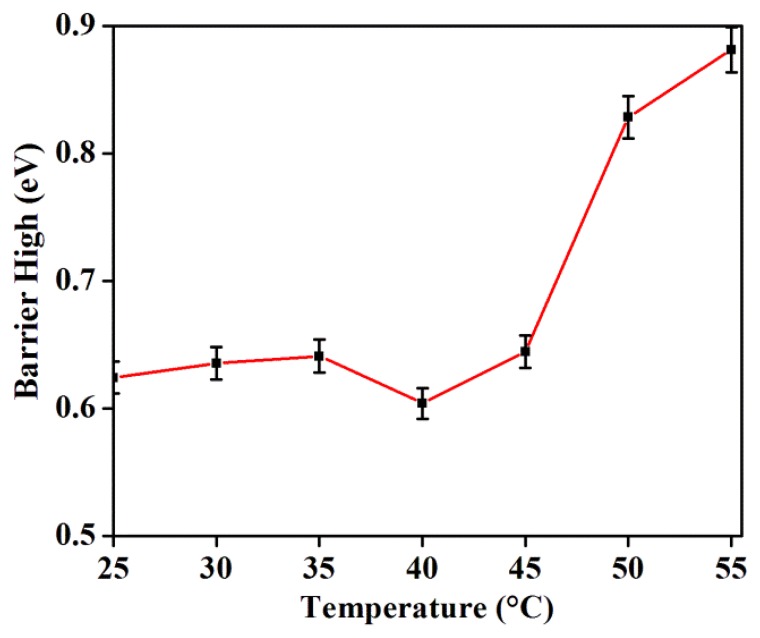
Temperature dependent potential barrier height in the absence of magnetic field.

Figure 6 illustrates the variation of GDG junction potential barrier height as a function of temperature calculated using the I-V curves. Up to a temperature of 45 °C the impact of the thermal field on the barrier potential is nominal, except for a slight decrease. However, the influence of temperature on the barrier height becomes significant beyond 45 °C. The observed temperature- mediated decrease in potential barrier height is in agreement with the increase in saturation current.

**Figure 7.**
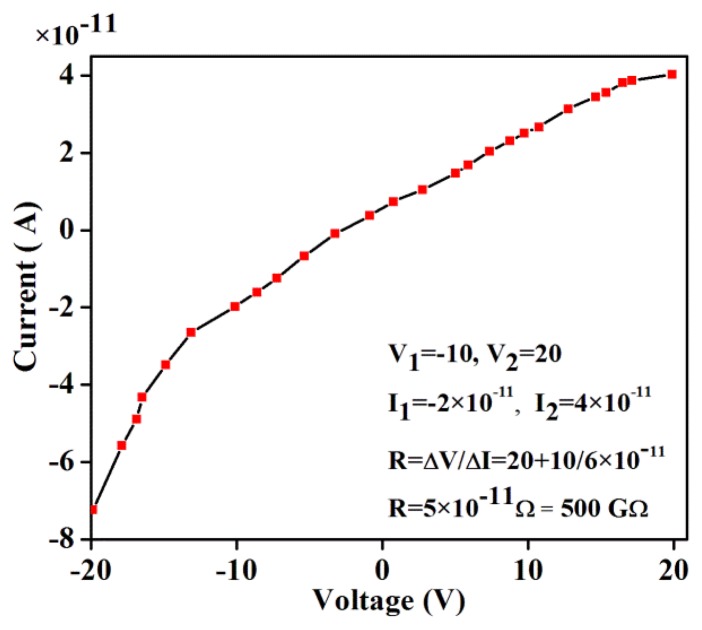
I-V curve of the junction between the two electrodes in the absence of DNA molecules.

A stronger magnetic field was created by bringing the magnetic poles closer and the applied magnetic field was calculated. In this case, the cryostat was removed from the device set-up and a magnetic field (200 mT to 1200 mT) was applied to the sample. To examine the effect of connectivity, I-V characteristics were measured in presence of magnetic field. Figure 7 shows the I-V curve for connection of Au-gap-Au at the absence of DNA. Resistance (500 GΩ) between the electrodes was significant without DNA, which produced a current of ∼10^−11^ A. The value obtained without the magnetic field showed no difference. It means that in this situation, the magnetic field does not have any effect on the sample. Upon placing DNA in the gap between the metals and applying a magnetic field, the I-V curve was obtained and analyzed. Simultaneously, the experiment that was performed without DNA as a control did not reveal the passage of any current.

The value of the room temperature potential barrier height calculated using the metal-semiconductor contact equation is found to be 0.75 eV, which is somewhat larger than the one obtained from the I-V curve. This higher value of potential barrier height is interpreted as follows: the rise in temperature increases the thermionic emission current which in turn enhances the possibility of carriers crossing through the barrier. In fact, temperature is directly correlated with the density of carriers and the occurrence of enhanced current is due to the increase in the number of carriers. Nevertheless, this increase in carrier density does not modify the actual length of the band gap but bends the potential barrier at the interface junction between metal and semiconductor. Consequently, it is the effect of temperature on saturation current that lowers the potential barrier compared to the actual value.

**Figure 8.**
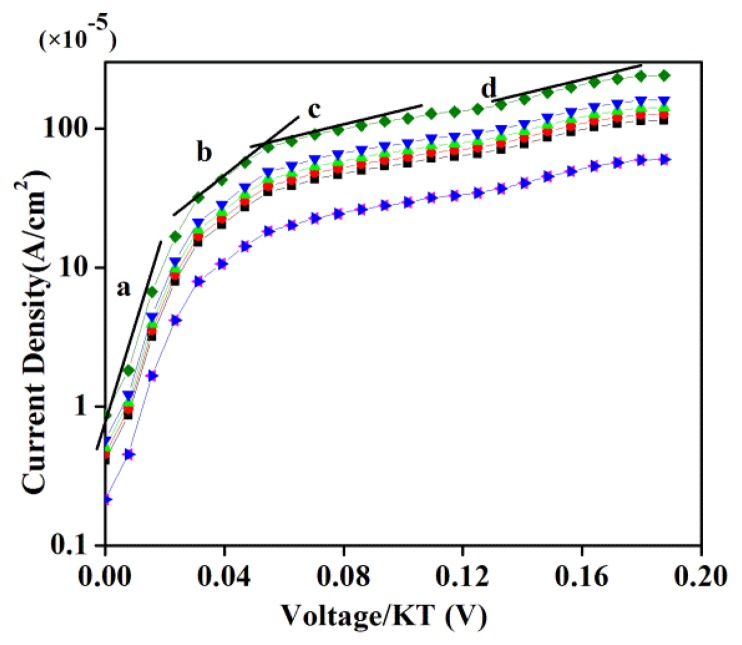
Temperature dependent current density without applied magnetic field.

Figure 8 presents the forward bias semi logarithmic I-V diagram at different temperatures, where k_B_T is the thermal energy and k_B_ is the Boltzmann constant. It is evident that beyond 45 °C the GDG structure does not reveal diode behaviour due to the structural alterations in the DNA at higher temperatures, where a larger resistance to current is manifested. For an ideal diode under forward bias, lines can be drawn on the curve at the point where the slope changes (as shown) to divide the diagram into several parts. A region obeying ideal diode behavior produces a diode emission coefficient of 1. In our case four regions are marked as *a*, *b*, *c* and *d*. The Schottky behavior is removed from the ideal diode state. In region *a*, the generated current deviates from diode performance due to recombination of carriers and the ideality factor becomes greater than 1. Conversely, the ideality factor in region *b* is calculated to be 1, while in region *c*, due to the penetrated diffusion of carriers this factor increases and deviates from normal diode performance. In the higher region *d*, the Ohmic effects of resistances lead to a complete deviation from diode performance. This departure from idealistic features became prominent with the increase of temperature. The evidenced radical change in current through the GDG structure at 50 and 55 °C is majorly attributed to the materials phase transformation and subsequent emergence of new crystal structures resulting from ruptured DNA.

**Figure 9.**
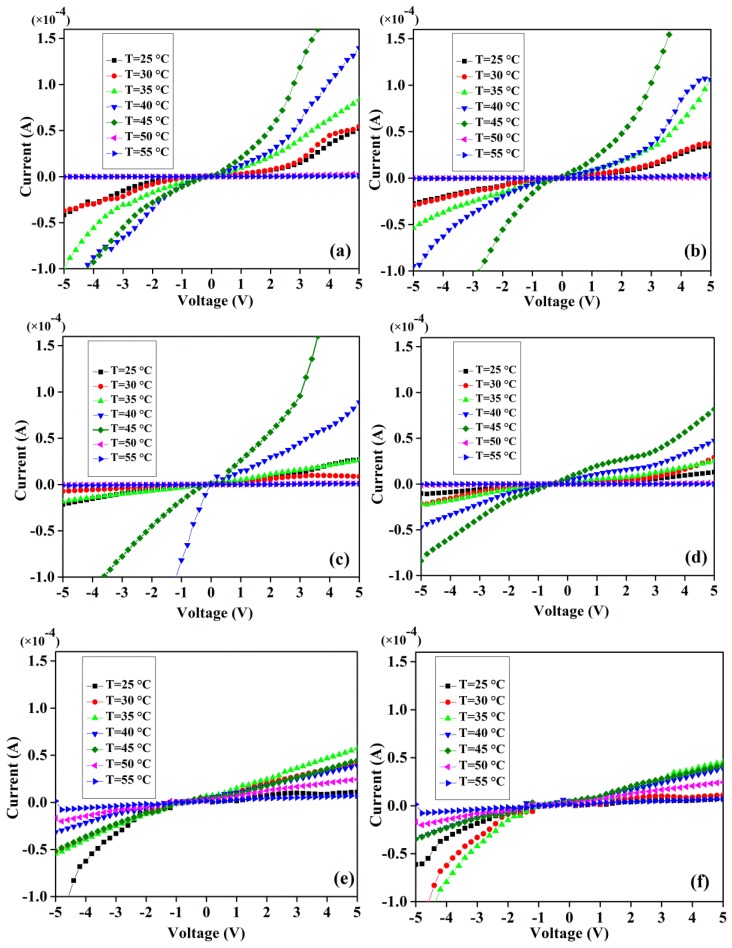
Temperature dependent I-V curves for: (**a**) 200 mT, (**b**) 400 mT, (**c**) 600 mT, (**d**) 800 mT, (**e**) 1000 mT and (**f**) 1200 mT applied magnetic fields.

Figure 9a–c display I-V curves at different temperatures under constant magnetic fields (200–1200 mT) as indicated. The effects of external magnetic field on potential barrier and Richardson constant are inspected using these characteristics.

**Figure 10.**
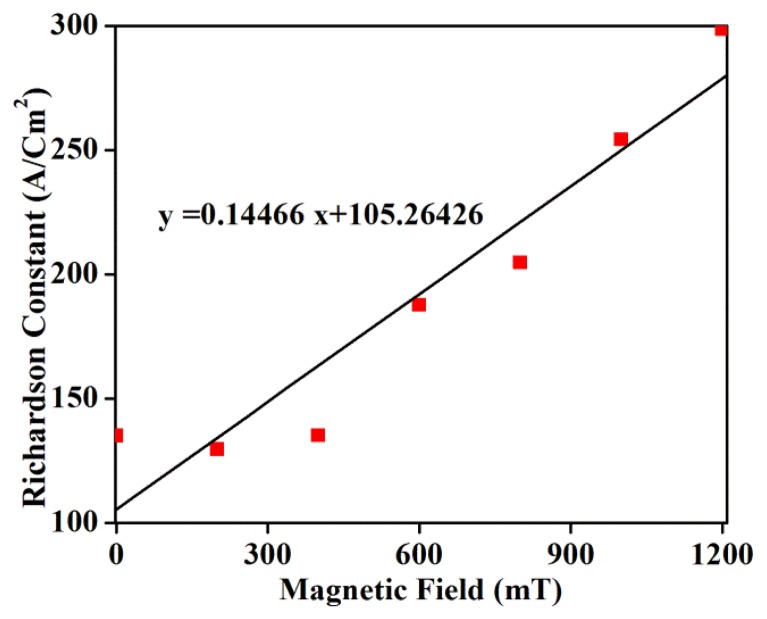
Magnetic field dependent Richardson constant for GDG structure.

Figure 10 shows that the Richardson constant is highly sensitive to the magnetic field variation. The Richardson constant, being a direct measure of the thermionic emission probability (heat-induced flow of charge carriers) from a surface or over a potential-energy barrier, is used to evaluate the diode performance of GDG structures. The thermal energy supplied to the carrier overcomes the binding potential, called the work function of the metal, before contributing to the current. The observed increase in the Richardson constant with the increase in magnetic fields is primarily ascribed to the increase in carrier effective mass and decrease in the mobility. This increase in effective mass causes a lower rate of carrier penetration through the potential barrier and thereby reduces the current.

Due to the increased rate of collision the number of electrons passing from the left to the right electrode is decreased and the resistance is increased. Hence, a decrease in current and increase in the potential barrier is observed. Calculated potential barrier heights using metal-semiconductor contact equation as summarized in Table 2 do not display any sizeable change for magnetic fields of less than 1000 mT. However, at higher magnetic fields the potential barrier is substantially enhanced.

## 4. Conclusions

The influences of external magnetic fields (up to 1200 mT) on the I-V characteristics of GDG structures with varying temperatures (25–55 °C) are determined experimentally. The I-V curves revealed semiconducting diode-like behaviour of DNA in GDG structures. With increasing temperature the current exhibited monotonic increases up to 45 °C at various voltages and suddenly dropped thereafter. This behaviour is interpreted in terms of the breakage of hydrogen bonds in the base pairs of the DNA macromolecular structure, which results an increase in the junction resistance. The decrease in potential barrier caused an increase in the saturation current with the increase in temperature. The increase in the Richardson constant with the increase of magnetic fields is due to the increase in carrier effective mass and decrease in the mobility. A correlation between the diode-like behaviour of GDG structure and magnetothermal effects is established. These new observations on the enhancements in the potential barrier, Richardson's constant and the resistance that cause a reduction in the current in GDG structures due to the combined effects of magnetic fields and temperatures may contribute towards the development of DNA-based magnetic and thermal sensors.

## Figures and Tables

**Table 1:** Relevant DNA parameters.

**DNA Sample**	**CG (%)**	**Melting Point (°C)**	**Molecular Weight (g/mol)**	**Length (nm)**
A (22%), T (20%), G (35%), C (23%)	58	87.5	176,975.1	567
bp DNA sequence	GGAGAATAACAAGGGTGCACGTGTGATGGTGGTGTGCTCCGAGCTCAACGTGATGTTCTTCCGTGGGCCTGACGACCACCACTTTGAGAACCTTATCGCACAAGCCCTCTTCGGCGACGGTGCTGCGGTGGTGATTGTCGGTGCAGGCCCAAAGGAGACAGAGAGACCGATCTACGAAGTGGCCTCGGCAGCACAGGTGATGCTGCCAGAGAGCGAGGAGATGGTTGCAGGGCACCTGAGGGAGATCGGGTTGACATTCCACTTAGCGAGTAAACTGCCGGCTGTTGTTGGCGCGAACATCCAACGGTGCCTGGAGGTGTCTTTCGCGCCAATGGGGGTTTCAAACTGGAACGAGCTATTCTGGATTGTGCACCCAGGCGGGAGAGCCATTGTGGACCAAGTTGAAATGAGTGCCGGGCTGGAGGCAGGGAAGCTAGCCGCGACTAGGCATGTGCTGAGGGAGTATGACAACATGCAGAGTGCTTCAGTGCTATTCATCATGGACGAGATGAGGAAGCGGTCGGTGGCAGAGGGATGCACCACCACCGGCGACGGCTTCGACTGGGG			

**Table 2.** Magnetic field dependent potential barrier height (*V_b_*) of Au-DNA-Au structure [6].

***B*** **(mT)**	***V****_b_* **(eV)**
0.00	0.883
200.00	0.880
400.00	0.882
600.00	0.890
800.00	0.886
1000.00	1.156
1200.00	1.175
